# Microalgae n-3 PUFAs Production and Use in Food and Feed Industries

**DOI:** 10.3390/md19020113

**Published:** 2021-02-18

**Authors:** Marine Remize, Yves Brunel, Joana L. Silva, Jean-Yves Berthon, Edith Filaire

**Affiliations:** 1 GREENSEA, 3 Promenade du Sergent Jean-Louis Navarro, 34140 MÈZE, France; marineremize@greensea.fr (M.R.); yvesbrunel@greensea.fr (Y.B.); 2 ALLMICROALGAE–Natural Products, Avenida 25 Abril, 2445-413 Pataias, Portugal; joana.g.silva@allmicroalgae.com; 3 GREENTECH, Biopôle Clermont-Limagne, 63360 SAINT BEAUZIRE, France; jeanyvesberthon@greentech.fr; 4 ECREIN Team, UMR 1019 INRA-UcA, UNH (Human Nutrition Unity), University Clermont Auvergne, 63000 Clermont-Ferrand, France

**Keywords:** microalgae, n-3 PUFA, EPA, DHA, food industry, feed industry

## Abstract

N-3 polyunsaturated fatty acids (n-3 PUFAs), and especially eicosapentaenoic acid (EPA) and docosahexaenoic acid (DHA), are essential compounds for human health. They have been proven to act positively on a panel of diseases and have interesting anti-oxidative, anti-inflammatory or anti-cancer properties. For these reasons, they are receiving more and more attention in recent years, especially future food or feed development. EPA and DHA come mainly from marine sources like fish or seaweed. Unfortunately, due to global warming, these compounds are becoming scarce for humans because of overfishing and stock reduction. Although increasing in recent years, aquaculture appears insufficient to meet the increasing requirements of these healthy molecules for humans. One alternative resides in the cultivation of microalgae, the initial producers of EPA and DHA. They are also rich in biochemicals with interesting properties. After defining macro and microalgae, this review synthesizes the current knowledge on n-3 PUFAs regarding health benefits and the challenges surrounding their supply within the environmental context. Microalgae n-3 PUFA production is examined and its synthesis pathways are discussed. Finally, the use of EPA and DHA in food and feed is investigated. This work aims to define better the issues surrounding n-3 PUFA production and supply and the potential of microalgae as a sustainable source of compounds to enhance the food and feed of the future.

## 1. Algae, a Source of Bioactive Compounds

The term “algae” describes a diversity of micro and macro aquatic organisms able to survive and proliferate by photosynthesis [1]. Macroalgae (multicellular) and microalgae (unicellular) develop in both freshwater and marine environments [1]. Microalgae are planktonic or benthic algae floating in the water, while macroalgae are benthic and sedentary [2,3]. They include either eukaryotic species or prokaryotic cyanobacteria that are devoid of a membrane-bound nucleus and are classified between bacteria and plants [1]. The most abundant microalgal phyla are blue-green algae (*Cyanophyceae*), green algae (*Chlorophyceae*), *Bacillariophyceae* (including diatoms), and *Chrysophyceae* (including golden algae) [1,4].

Microalgae represent an exciting competitive source of biomass, especially because of their very efficient photosynthetic system. Indeed, thanks to their aqueous environment and submerged nature, microalgae have continuous access to water, CO_2,_ and nutrients that allow them to be very effective in converting energy to biomass [3,5]. They are also easy to produce and can develop under various trophic regimes. Like terrestrial plants, most microalgae are autotrophic organisms efficiently using light to grow [6]. However, some species, for example, those belonging to the phylum Dinoflagellata, can use organic molecules to grow in addition to the inorganic ones and are called mixotrophic [7]. Heterotrophic microalgae, such as the Dinoflagellate *Crypthecodinium cohnii*, also exist, and exclusively use organic sources like glucose for carbon metabolism and energy [3]. This is also the case with marine protists from the Thraustochytrid family such as *Schizochytrium*. Even if they cannot be considered algae as they lack plastid and chlorophyll, these organisms are exclusively heterotrophic [8,9]. Microalgae are also very diverse, and more than 30,000 species have been described so far [10,11]. The ease with which they can be cultured explains in part why they have been receiving increasing interest in recent years. Microalgae chemical composition has been widely studied in the literature, with proportions varying widely between the different species and culture conditions used [1,12]. They have been recognized as a reliable source of bioactive compounds such as proteins, lipids, carotenoids, hydrocarbons and vitamins [3,6]. The levels of these compounds of interest in microalgae are similar or even higher than they are in plants and animals [5,6]. The diversity of the compounds associated with the critical number of species available makes it possible to select and choose specific strains and molecules for various applications. These molecules also exhibit very interesting properties such as antioxidative, anti-microbial, anti-inflammatory, coloring, texturing, stabilizing, or even emulsifying capacities, which explains why they are so widely valued in the food and feed industry, in the cosmetics and medical sectors, and for bioenergy, biodiesel or even aquaculture [3,6,12]. 

In recent years, particular attention has been given to microalgae lipids. Indeed, they can represent up to 74% of microalgae’s total biochemical content according to species [12]. These molecules are built with fatty acids with 12 to 24 atoms of carbon and include the polyunsaturated fatty acids of the n-3 or n-6 families (n-3 PUFAs and n-6 PUFAs, respectively) [1]. N-3 PUFAs, also called omega-3 fatty acids, are characterized by their health benefits for both animals and humans. Their properties are expected to be of central interest in developing novel ingredients or compounds for various sectors, including the feed and food industry, with high commercial value. So far, microalgae are still poorly explored and valued as a natural source for a healthy diet [13]. Among the critical number of species known to date, only a few are commercially produced due to strict food safety regulations, namely in Europe, and because their cultivation in industrial quantities is only a few decades old [10,11]. This translates into a high potential for the production and commercialization of these organisms in the coming years.

Thus, this review aims to focus on the future challenges of n-3 PUFA production by microalgae and its availability and role for humans and aquaculture in the context of stock reduction and global warming. For this purpose, the first part includes a review of the background situation concerning overfishing and environmental changes. Then, the production of n-3 PUFAs by microalgae is developed, and the current knowledge on their biosynthetic pathways is discussed. Finally, examples of the application of these healthy compounds in the food and feed industries are presented to help better foresee the future of this field in the years to come.

## 2. Polyunsaturated Fatty Acids and Human Health

Human consumption of seafood has been increasing for several years, mainly due to seafood’s richness in protein, n-3 PUFAs, vitamins and minerals, and its well-known health benefits [14]. In fact, marine products are one of the best food sources of n-3 PUFAs, also called omega 3s. At the bottom of the marine food chain, microalgae can produce EPA and DHA from smaller polyunsaturated fatty acids, linoleic acid (LA—18:2n-6) and α-linolenic acid (ALA—18:3n-3), due to a series of dedicated desaturases and elongases. However, unlike these organisms, humans do not possess these enzymes and are unable to produce EPA and DHA in sufficient quantities [15]. Both fatty acids are known for their therapeutic properties with regard to cardiovascular diseases, hypertension, and autoimmune disorders [14,16]. They are therefore of the most importance for humans and need to be included in their daily diet [17].

Microalgae are known to synthesize large quantities of PUFAs, a variable amount of monounsaturated fatty acids (MUFA) and low amounts of saturated fats (SFAs) [18]. Dietary intakes of n-3 PUFAs, particularly eicosapentaenoic acid (EPA—20:5n-3) and docosahexaenoic acid (DHA—22:6n-3) have long been known to reduce the risk of developing cardiovascular problems [19,20].

### 2.1. n-3 PUFAs and Cardiovascular Diseases

Numerous studies have been conducted in past years to understand how these nutrients can act on cardiovascular disorders. Research is ongoing and sometimes appears incoherent. Indeed, while early trials attested to beneficial cardiovascular outcomes, recent works are nuanced [21]. Difficulties in authenticating the impact of these two PUFAs stem from the heterogeneous nature of the population tested, the absence of standardized clinical trials to evaluate their actions, varying sample sizes, the omega-3 doses tested, and the EPA to DHA ratio [22]. For example, conflicting results on the impact of EPA, DHA, and a combination of both on myocardial infarction have been observed [23]. EPA and DHA used individually (5 g × day^−1^) seemed to diminish infarct size, while a combination of both to a level of 5 g × kg^−1^ did not [23]. Supplementation of EPA+DHA to a level of 1.8 g/day^−1^ for elderly patients with a recent acute myocardial infarction did not reduce clinical events either [24]. This might be due to n-3 PUFAs’ competition for the same receptors or biased signaling as observed with some protein receptors [23]. Generally, the available data seem to support the positive impact of dietary n-3 PUFAs on blood pressure [20], congestive heart failure [25], and supplementation of more than one g/day^−1^ of n-3 PUFAs seem effective in lowering the risk of fatal cardiac events [21]. Thus, further studies are warranted to better define these nutrients’ role and activity in cardiovascular disease development and prevention.

### 2.2. n-3 PUFAs Against Obesity and Diabetes

EPA and DHA can also prevent obesity by inhibiting certain enzymes responsible for lipid synthesis, affecting serum lipids and lipoproteins [14]. In animal models, omega-3 supplementation improved hepatic insulin sensitivity, the production of adipocytokines and direct and indirect anti-inflammatory effects [22]. For example, omega-3 supplementation can prevent diabetic complications such as diabetic nephropathy connected to renal phospholipid metabolism in rats, and n-3 PUFAs and MUFAs phospholipid synthesis is enhanced, and linked to anti-inflammatory mechanisms [26]. For humans, the mechanisms might vary as a function of genetics and/or lifestyle [22]. Insulin sensitivity seems to increase after supplementation of n-3 PUFAs in relation to non-esterified fatty acid reduction [27]. EPA and DHA prescription for Type 2 diabetes mellitus significantly reduces the levels of triglyceride known to be responsible for fat accumulation, thereby reducing the risk of developing hypertriglyceridemia [28]. However, the EPA-only prescription seems slightly better as it does not increase the concentration of low-density lipoprotein cholesterol, decreases total cholesterol levels, and results in fewer gastrointestinal effects than DHA-only prescription [28]. Interestingly, these n-3 PUFA-reinforced diets do not result in glucose metabolism impairment in diabetic patients, as shown in 14 clinical trials [29].

### 2.3. Anti-Inflammatory Properties of n-3 PUFAs in the Context of the COVID-19 Pandemic 

EPA, 20:5n-3, is a precursor of eicosanoids and can limit inflammatory developments related to chronic diseases [16,30]. Both EPA and DHA can regulate the production of bioactive substances such as eicosanoids [15]. Increasing the ratio of n-3 to n-6 PUFAs decreases inflammation, as omega-6 is a precursor of pro-inflammatory molecules while eicosanoids issued from omega-3 have antagonist properties [14]. N-3 PUFAs can alter the decomposing enzymes and inflammatory factors or up-regulate the activities of some proteins or enzymes such as lipoprotein lipase fatty acid-binding protein [15]. More precisely, mechanisms underlying the anti-inflammatory actions of EPA and DHA involve inhibition of leukocyte chemotaxis, reduction of adhesion molecule expression and leukocyte-endothelial adhesive interactions, disruption of lipid rafts, inhibition of activation of NF-κB, activation of anti-inflammatory transcription factors, such as Peroxisome Proliferator-Activated Receptor Gamma (PPARγ), and binding to the G protein-coupled receptor (GPCR120) [30]. It is also important to note that pro-resolving mediators, such as resolvins, protectins, and maresins synthesized through the enzymatic oxidation of EPA and DHA, involve the COX and LOX pathways, and are inflammation-resolving, inhibiting transendothelial migration of neutrophils and cytokines (IL-1β and TNF-α), and chemokines production. N-3 PUFAs also increase phagocytotic capacity, decreasing the reactive oxidative species (ROS) of innate immune cells, including macrophages and neutrophils. Moreover, n-3 PUFAs and their metabolites also promote activation of NK cells and modulate T cell activation by altering the activation of antigen-presenting cells (APCs, such as macrophages or dendritic cells) and prevent the differentiation of CD4+ cells to Th1 cells. This anti-inflammatory effect appears all the more important in the current context of the global pandemic. Even if there is no known definitive treatment for SARS-CoV-2 induced COVID-19, antiviral and supportive treatments are essential for patients with COVID-19. As cytokine storm is a very common manifestation in severe patients and often leads to exacerbation, intervention with anti-inflammation therapy may help in preventing further injury [31]. Recently, Torrinhas et al. [32] reported that immune modulatory properties of EPA and DHA provided in emulsions might play a key role in changing clinical outcomes of SARS-CoV-2 infected patients. Even if the benefits expected based on the anti-inflammatory activity of EPA and DHA are anecdotal and need to be verified by rigorous clinical trials, they suggest that a prescription based on body weight (e.g., 0.2 g pure fish-oil lipid emulsions/kg body weight/day), combined with low oral aspirin intake to trigger resolvin synthesis from EPA and DHA, could be beneficial to patients [32].

### 2.4. EPA and DHA Roles in Gut Microbiota 

Omega-3 supplementation has also been proven to impact the development and composition of gut microbiota positively. The intestinal microbiota is involved in immune response and prevention of pathogenic invasions [33]. They can also stimulate the resident macrophages, for example, via the production of tryptophan and indole-3-acetate [34]. Both can help in eliminating the pro-inflammatory molecules induced by lipopolysaccharides or palmitate. The composition of gut microbiota is influenced by phenotype, age, gender, exercise frequency, dietary habits, the use of antibiotics, drugs, probiotics, immune function, geographical location, environment, and host-specific in humans [35]. Any alteration of the gut microbiota or imbalance between microbial communities, called dysbiosis, might be responsible for metabolic or neurologic disease development [35]. Diet is one of the main factors pressuring microbial communities. The nature of dietary fats influences the types and number of gut microbes and the host’s intestinal health [36]. The communities present determine the level of resistance to infection and susceptibility to inflammatory diseases. Some communities’ changes have been recorded following the incorporation of alimentary fats including n-3 PUFAs [36]. For example, diets enriched with n-3 PUFAs for high-cardiometabolic-risk patients seem to be associated with modification of lipid metabolism with the promotion of *Bifidobacteria* that produces butyrate, an essential metabolite for the human colon [37]. N-3 PUFAs are also thought to have positive actions on gut microbiota. As an illustration, Bentley-Hewitt et al. [38] studied the interactions between *Lactobacillus* sp. and n-3 PUFAs. They showed that direct exposure of *Lactobacilli*, a prominent community of intestinal bacteria, to ALA, EPA, and especially DHA could enhance their probiotic potential, as these nutrients improved their abilities to adhere to the epithelial layer. Furthermore, the health benefits of EPA and DHA, such as their antimicrobial activities, can be triggered by intestinal microbiota’s enzymatic actions [36].

### 2.5. Daily Recommendation and Requirement of n-3 PUFAs

Accordingly, European authorities have established their recommendations based on epidemiological studies attesting to the beneficial effects of a Mediterranean-style diet [39]. This diet consists of high fat intake (40–50% of total daily calories) with less than 8% of saturated fats and 15 and 25% of monounsaturated fatty acids. A higher consumption of n-3 PUFAs than n-6 PUFAs is also advised in addition to a low ratio of n-6:n-3 comprised between 2:1 and 1:1 [39]. Because of the rising health concern for humans, there is an urgent need to target food and bioactive compounds that could meet the requirements of n-3 PUFAs for general health [40]. Health agencies’ recommendations on the daily consumption of these two fatty acids have been stated [41]. A worldwide consensus (World Health Organization, Europe, USA, Australia, New Zealand, United Kingdom, Netherlands, France, Canada and Japan) has been reached in recent years, recommending between 250 and 500 mg of EPA+DHA per day. More specifically for France (ANSES), the recommendation has remained unchanged since 2011 at 500 mg × day^−1^ EPA+DHA (250 mg.day^−1^ each) [42]. It is advised to consume at least two servings of fatty fish per week to ensure good health and reduce the risk of mortality from cardiovascular diseases [43].

### 2.6. Role of n-3 PUFAs on Brain Development, Tumors and Cancer

Polyunsaturated fatty acids also have a positive impact on brain development. DHA is one of the main structural components of nerve cells and helps transmit signaling messages to maintain the plasticity of the brain [44,45]. Arachidonic acid (ARA—20:4n-6) is also involved in brain functioning and eases neuronal transmission and long-term potentiation [44]. They are both preserving it against oxidative stress. DHA, as a component of photoreceptor cells, is also indispensable for vision [44]. Rodent and human models show that DHA supplementation could not only have a beneficial impact on brain functions, including learning and memory [46,47], depressive and aggressive behaviors [45,48] but also on audition [49,50], and olfaction [51,52].

N-3 PUFAs also participate in synapse formation, neuronal proliferation, and differentiation and extension of nerve fibers [53]. DHA had also been studied as a valuable compound to delay brain aging and protect against the onset of Alzheimer’s disease (AD) [54]. Among those with other brain disorders, patients with Schizophrenia might also present low levels of n-3 PUFAs and ARA in their body tissues [55] and research suggests that treatment including supplementation with EPA and/or DHA could decrease violence in schizophrenic patients [56].

EPA and DHA also have antitumoral and anticancer properties. Indeed, EPA and DHA can improve drug delivery by modifying the tumor vasculature with architectural remodeling [57], and present antiangiogenic activities [58,59]. They also induce apoptosis [60,61], block the cell cycle resulting in stopping tumor development [53], and participate in lipid peroxidation-mediated endoplasmic-reticulum-stress which triggers deterioration of cancer cells [62].

### 2.7. N-3 PUFAs and Retinopathies

PUFAs and their derivatives can also play a role in the pathogenesis of retinal diseases (retinopathies) like retinopathy of prematurity (ROP), age-related macular degeneration (AMD), or diabetic retinopathy (DR). They are essential nutrients for the visual system, and DHA is a major structural component of retinal photoreceptors [63]. Due to their anti-angiogenetic proprieties, they can reduce retinal stress in ROP [64], their anti-oxidant capacities they can protect eye photoreceptors from oxidative stress [65]. N-3 PUFAs can avert visual loss owing to AMD [63,66]. Lipid metabolism impairment in diabetic patients can also impact the retinal system [58]. Hyperglycemia triggers various pathological alterations such as oxidative stress, inflammation, angiogenesis, increase of apoptosis of endothelial cells and neurons, and injury to the retinal blood capillaries [67]. Thus, with the previously described effects of omega-3 fatty acids, including α-linolenic acid (anti-inflammatory, anti-oxidative or anti-angiogenic proprieties among others) on such disorders, they are of immense interest in preventing DR [68].

Thus, there is increasing interest around EPA and DHA for their positive effects on health for both humans and animals. This correlates to a continually increasing demand for these two compounds in recent years. It is thus imperative to find sustainable ways of supplying polyunsaturated fatty acids to meet this demand.

## 3. Increasing Demand for n-3 Polyunsaturated Fatty Acids 

A significant increase in fish and marine product consumption was registered between 1961 and 2016, with an average annual growth of 3.2% according to the FAO (2018) [69]. However, these trends are being challenged by supply issues [40]. Supply of n-3 PUFA comes mainly from the ocean and the vast majority (almost 90%) from capture fisheries [40]. Thus, access to fish and seafood determines to what extend the supply can meet PUFA demand. Global fisheries have reached a plateau following stock reduction (90 million tons per annum) [40,69]. Areas that were previously unexploited or underexploited in the 1950s are now undergoing an unprecedented expansion in fishing by industrial fleets [70]. Exploitation in the Southwestern Atlantic, the Indian Ocean, and the Western Central and Southwest Pacific has peaked [70]. These demanding industrial fisheries are responsible for removing top predators and thus the release of predatory pressure on lower trophic levels [70]. Pauly et al. (1998) [71] observed a decline in mean trophic levels. They proposed the concept of “fishing down marine food webs” which illustrates how the intensive capture pressure exercised on top predators leads to a change of target for lower trophic levels. Indeed, the diminution of top-down predatory pressure on smaller fish leads to a subsequent increase in their biomass, thus promoting new intensive captures by fisheries [70]. Overfishing is also responsible for stabilizing fish harvesting in the last few decades (33.1% of stocks were overfished in 2018) [69]. Most fisheries are at or beyond their sustainable levels of exploitation [40]. Technological improvement in fishing gear keeps increasing catching efficiency [72]. The fishing power of fleets and their range of action and versatility are dramatically affecting small pelagic fish [70].

However, these small trophic levels, including phytoplankton are also affected by climate change [73]. Global warming is suspected to impact polyunsaturated fatty acids production by phytoplankton, thus reducing the availability of these essential compounds for higher trophic levels [74]. Temperature is expected to have effects on the quantity and quality of fatty acids in phytoplankton [74]. At the bottom of marine food webs, phytoplankton can adapt to changing temperatures by modifying their membrane characteristics [75]. Indeed, lipids and especially fatty acids are critical elements of cell membranes and can be modulated in response to environmental changes to maintain the desired level of fluidity [73,76,77]. The unsaturation of PUFA enhances the fatty acid’s ability to fold and increase flexibility [74]. Generally, n-3 PUFAs decrease with increasing temperature, while n-6 PUFAs have antagonist dynamics with rising temperature [74]. The increase in water temperature may then induce major shifts in global PUFA production by phytoplankton and their first consumers, microzooplankton, which could then have reverberating effects on higher terrestrial and aquatic trophic levels [18,74].

Consequently, considering the constant growth of the global population and the resulting demand for these compounds, aquaculture now appears to be one of the solutions for meeting future seafood needs and filling the gap between supply and demand [40,78]. World aquaculture’s contribution to world fish production increased to 46% in 2016–2018 compared to only 26% in 2000 [79]. It is the first source of fish and ensures a continuing rise in fish supply for human consumption [69]. Global aquaculture production in 2018 consisted of 82.1 million tons of aquatic animals, 32.4 million tons of aquatic algae, and 26,000 tons of ornamental seashells and pearls [79]. Unfortunately, aquaculture remains mostly dependent on other fisheries’ products such as fish meal or fish oils (22 million tons in 2018) [78,79]. Consequently, finite and limited fisheries of small pelagic species like anchovy or sardine are more harvested, exacerbating pressure on fish stocks and significantly increasing sales prices [79]. In the longer term, aquaculture thus appears not to be economically sustainable, especially since climate change already impacts the natural production of these oils.

The new task is to find alternative n-3 PUFA sources and more sustainable fish oils and meal origins able to supplement aquaculture production and capture fisheries. One of such alternatives is the use of lower trophic level species like zooplankton, krill or copepods. This option is challenging in terms of harvesting and raises environmental questions on higher trophic levels. Top predators like whales and penguins are directly relying on these groups to survive. By collecting the prey this alternative might impact the predators, and then not be sustainable in the longer term [72]. Utilization of higher plants as a substitute for aquaculture feed is conceivable [72,80], however since they are not able to synthesize 20:5n-3 and 22:6n-3 due to the absence of dedicated enzymes [78,81], they would only supply precursors (like 18:2n-6 or 18:3n-3) and be difficult to promote for n-3 long-chain PUFAs (LC-PUFA) production by aquacultured fish. Consequently, the production of EPA and DHA by higher plants is currently attempted to implement genes of organisms naturally producing these compounds, i.e., microalgae, yeast and/or bacteria [78].

Developed in the mid-1970s for use on common crops, genetic engineering has been adapted in recent years, thanks to improvement in techniques, more specific DNA-markers and the use of genomics [82]. Genetic modifications have already introduced the n-3 PUFA biosynthesis trait to oleaginous plants. For example, this genetic engineering has been tested on *Nicotiana tabacum* [83], *Arabidopsis thaliana* [84], *Brassica juncea* [85], *Cannabis sativa*, and *Camelina sativa* [86,87]. At first, these transgenic plants could not match the level of EPA and DHA of microalgae or the requirement for farmed fish [78], especially in DHA [81]. However, recent studies have shown more promising results, proving, for example, the efficiency of *Camelina* crops to substitute both fish and vegetable oil without any negative effects on fish development or health [38,87,88]. They are real alternatives to less sustainable aquafeeds satisfying the demand for essential fatty acids as well as increasing 20:5n-3 and 22:6n-3 content for the human diet [40,88]. However, these genetically modified plants are often negatively regarded by consumers as artificial and unnatural and are perceived to carry risks for health and the environment [89]. Industries have to work on their transparency and communication strategies in order to increase the acceptance of such products on the market.

Another encouraging solution is the direct production of microalgae rich in these two PUFAs. Algal biomasses are currently used and formulated for farmed fish feed and human consumption and are commercialized by different industries [40]. These aspects will be developed later on in this review. However, even if the fatty acid composition of the different microalgae species is widely studied [76,90,91,92,93,94,95], more knowledge is needed to elucidate their synthesis pathways, as they will be particularly useful in developing new sustainable products for aquafeed as well as for human food.

## 4. Microalgae as n-3 Polyunsaturated Fatty Acids Producers

As already stated above, humans or top predators cannot synthesize de novo n-3 PUFAs or produce them from their precursors in large enough quantities. They thus have to obtain them from their diet [96,97]. It is well accepted that the main sources of LC-PUFAs are microalgae. Their average lipid content varies from between 1 and 40% according to the species and growth conditions [5]. Fatty acids that cannot be synthesized by all organisms are considered essential fatty acids [98]. This is the case for linoleic acid, α-linolenic acid or even longer PUFAs such as EPA, DHA or ARA [97].

The percentage of 20:5n-3 and 22:6n-3 varies with microalgae species and depends on the productivity rate and the growth conditions imposed [96]. Microalgae richer in EPA and DHA consequently present a higher nutritional value for consumers than microalgae with lower n-3 PUFA levels [96].

DHA-producers are known to be Dinophytes, Haptophytes, some Cryptophytes, Thraustochytrids, or Euglenoids [92,93,97,99,100]. Dinophytes can produce high amounts of 22:6n-3 with up to 40% of the total fatty acids in some taxa [99,101,102]. In Haptophytes, DHA production can reach 30% of total fatty acids [103]. Thraustochytrids remain some of the most essential 22:6n-3 producers and synthesize around 60% of total fatty acids in the form of triacylglycerides (TAG) in the genus *Aurantiochytrium* and *Schizochytrium* [100,104].

EPA-synthesizing taxa are Diatoms, such as *Phaeodactylum tricornutum*, Eustigmatophyte such as *Nannochloropsis* sp. and some Haptophytes [97,105,106,107,108,109,110,111]. Diatoms can synthesize around 20% of EPA and a low proportion of DHA [109]. The Eustigmatophyte, *Nannochloropsis oculata*, produces between 15 and 30% of 20:5n-3 [110]. 

For other taxa such as Cyanobacteria or Chlorophytes, long chain n-3 PUFA production can be assumed negligible [96], and only certain levels of PUFAs such as ALA and stearidonic acid (SDA—18:4n-3) can be found and play a subsiding role in consumer development, namely *Chlorella vulgaris* [97].

In addition to inter-species variability, LC-PUFA production can also be modulated by environmental parameters and varies with phytoplankton growth. Factors such as temperature, growth rate, irradiance, salinity, and nutrient availability can control phytoplankton PUFA production. Indeed, as stated earlier in this review, microalgae can modify their membrane fluidity in response to temperature variations, thus adapting their fatty acid composition [76,77]. Anterior works studied the impact of nitrogen and nutrient availability and showed that microalgae tended to accumulate neutral lipids when nutrients became scarce [112,113]. Microalgae sensitivity varies with taxa and can also have a tremendous impact on this lipid accumulation intensity following environmental stress [112]. In contrast, phytoplankton’s exponential growth phase associated with replete nutrient conditions would be more likely linked to higher production of polar lipids used to build cell membranes [113]. Taipale et al. [97] studied the influence of the growth stage on the production of n-3 and n-6 PUFAs in 16 species belonging to the six main groups of phytoplankton (Cryptophytes, Dinophytes, Chrysophytes, Diatoms, Chlorophytes and Cyanobacteria). They showed that PUFA content can vary greatly within phytoplanktonic groups to variation in cell size. Both increasing temperature and concentration of nitrogen were related to the production of SDA, EPA, DHA and docosapentaenoic acid (DPA-6—22:5n-6) in Cryptophytes, Chrysophytes, and Dinophytes, and the PUFA production of some taxa was more favored during the stationary phase (Cryptophytes and Chrysophytes), while others would be during exponential phase (such as fast-growing diatoms and dinophytes) [97].

Despite the extensive knowledge of the diverse species able to produce these two compounds, the synthesis pathways leading to the production of EPA and DHA are still under investigation. They would be of vital interest in improving microalgae cultures and enhancing the n-3 PUFA yields for industries, especially for healthy food and feed preparations.

## 5. Synthesis and Production of n-3 Polyunsaturated Fatty Acids

### 5.1. Nomenclature 

Fatty acids are chains of hydrocarbons with a carboxyl group (COOH) and a methyl group (CH) on opposite sides of the molecule. These chains can vary in length, degree of unsaturation and ramification. Fatty acids without double bonds within the hydrocarbon chain are called saturated fatty acids. The mono-unsaturated fatty acids present only one double bond, while the polyunsaturated fatty acids possess several double bonds. These differences in length, location and numbers of unsaturation are used in the conventional nomenclature. The positioning of the first double bond relative to the methyl terminus carbon (“n”) is used to name fatty acids. SFAs are then noted X:0 with X the number of carbon atoms while MUFAs are annotated X:1n-Z and PUFAs X:Yn-Z with Y the number of double bonds and Z the position of the first double bond relative to the methyl terminus carbon, respectively [114]. Differences in the configuration are possible: a “cis” configuration indicates that carbon functional groups are on the same side of the carbon chain, while a “trans” configuration has functional groups at opposing sides of the carbon chain [114]. N-3 PUFAs, which include fatty acids such as EPA and DHA, are then polyunsaturated fatty acids presenting their first double bond on the third carbon from the methyl-end side of the carbon chain, while for n-6 PUFA (such ARA), the first double bond is located between the sixth and the seventh carbons.

### 5.2. Fatty Acid Synthase (FAS): Synthesis of Saturated Fatty Acids

The biosynthesis of fatty acids is similar among plants and animals. It is initiated by de novo synthesis of acetyl-CoA via the concerted action of acetyl-CoA carboxylase (ACCase) and fatty acid synthase [115]. The metabolic pathway involved in lipid synthesis is initiated by glucose. Glucose is metabolized into pyruvate, then decarboxylated and oxidized by the pyruvate dehydrogenase to produce acetyl-CoA. In microalgae, fatty acid biosynthesis is assumed to be similar to that of higher plants: acetyl-CoA and de novo fatty acid synthesis seem to take place in the plastid (chloroplast) and require energy (ATP) and reducing power (NADPH) [116]. Differences in morphology and physiology of microalgae species, their cellular organization and carbon metabolism is expected to impact lipid trafficking and synthesis pathway [116]. Little is known about the routes responsible for acetyl-CoA synthesis in microalgae, but like in plants, acetyl-CoA is assumed to have two origins: it can be provided by direct supply from plastid by pyruvate dehydrogenase/decarboxylase or indirectly from mitochondrial pyruvate dehydrogenase where the mitochondrial acetyl-CoA has to be transferred to the plastid after hydrolysis to be regenerated by ACCase [117].

**Figure 1:**
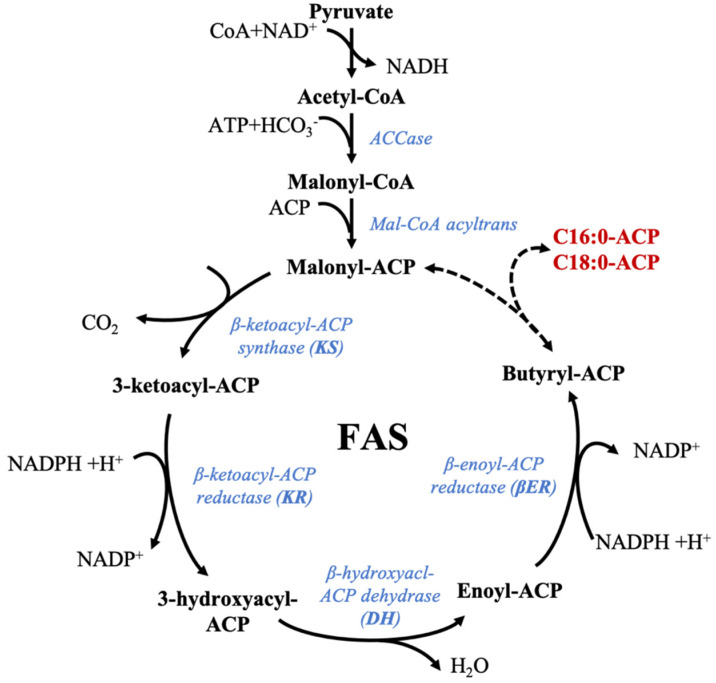
Fatty acid synthase (FAS). Fatty acid synthesis is initiated by malonyl-CoA. The different iteration of the cycle following the action of the four enzymes of the complex (KS, KR, DH, βER) add two atoms of carbon to produce saturated fatty acids such as 16:0 and 18:0. See the text for further details.

The first step of the fatty acid synthesis pathway is the reaction between acetyl-CoA and bicarbonate to form malonyl-CoA [116,117]. Malonyl-CoA has a central role in this synthesis as the initial precursor of the de novo fatty acids synthesis, but also by being useful in the later elongation steps occurring in the endoplasmic reticulum (ER). The conventional fatty acid synthesis pathway described in plants and microalgae is divided into three distinct pathways supported by three enzymatic systems: (i) first, the biosynthesis of palmitic acid (16:0) and other saturated fatty acids is completed from acetyl-CoA, following the Fatty Acid Synthase (FAS) pathway (Figure 1), (ii) then further chain elongations, and (iii) desaturations occur as part of the n-3 and n-6 pathways to produce more complex polyunsaturated fatty acids [116,117,118]. In microalgae, chain elongation and desaturation happen in the mitochondria and endoplasmic reticulum, desaturation steps in the endoplasmic reticulum, and de novo synthesis in the plastid [116]. Lipid chains are created from malonyl-CoA by successive additions of two-carbon units involving four enzymatic steps: condensation, reduction, dehydration, and another reduction and consuming NADPH while releasing CO_2_ [116]. In microalgae, fatty acid biosynthesis is performed by stromal fatty acid synthase (type II) which consists of a multisubunit composed of four monofunctional enzymes ensuring the following four enzymatic reactions [116]. The different reactions catalyzed by plant FAS and supposed to be identical for microalgae FAS as genes coding for these enzymes have been characterized for phytoplankton and were described earlier [116] (references therein). The condensation step is made by β-ketoacyl-ACP synthase (KS) and forms a simple carbon-carbon bond. Each type of KS is responsible for a different reaction: KSIII is in charge of the initial condensation between malonyl-CoA and acetyl-CoA, KSI is used during the six following iterative steps to produce 16:0-ACP, and the last elongation reaction of 16:0-ACP is conducted by KSII to form 18:0-ACP [117]. The first reduction reaction by β-ketoacyl-ACP reductase (KR) is NADPH-dependent. Next, the hydration step by β-hydroxyacyl-ACP participates in the synthesis of enoyl-ACP, which in turn is reduced by a second reductase (β-enoyl-ACP-reductase—βER) to form a saturated acyl-ACP [117]. Type I fatty acid synthase, present in mammal cytoplasms, consists of seven catalytic components linked together in a multifunctional megasynthase [116]. Its existence in microalgae cytoplasms remains unclear, but it could be possible, as putative FAS I enzymes have been identified in *Nannochloropsis oceanica* and *Euglena gracilis* [119,120]. The schematic representation of the supposed microalgae FAS pathway is available in Figure 1. Several iteration steps of the FAS pathways allow for the formation of palmitic acid (16:0).

### 5.3. Elongation and Desaturation Steps of the n-3 and n-6 Pathways

In microalgae and higher plants, at this point, palmitic acid can follow three different routes: (i) it can be used in the plastid to form chloroplast lipids such as the galactolipids, (ii), further elongated into 18:0 and then more complex fatty acids or (iii) converted into a free fatty-acid [121,122]. The next steps of the synthesis pathway allow the production of a panel of fatty acids varying in both chain length and degree of unsaturation. After being synthesized by the FAS pathway, 16:0 encompass one last condensation, reduction, dehydration and reduction step, respectively, and gives a saturated two-carbon longer fatty acid: stearic acid (18:0). 16:0 and 18:0 are the major products of the FAS pathway in microalgae [116]. An acyl-CoA desaturase is then involved in the following reaction step. A desaturase is a special type of oxygenase able to remove two hydrogens from a hydrocarbon chain, catalyzing the formation of a double bond in the substrate [121,123]. According to their regioselectivity, desaturases are noted Δx where x refers to the position from the carboxyl-end of the fatty acid where the double bond is added [123]. After FAS synthesis, a Δ9-desaturase (adding the double bond on the ninth carbon from the carboxyl-end) is responsible for the formation of the first double bond, leading to the formation of the first monounsaturated fatty acids, palmitoleic acid (16:1n-7—PAL) and oleic acid (18:1n-9—ODE) respectively, from 16:0 and 18:0 [116].

The fatty acids formed by FAS and from FAS-products following the action of desaturases have two possible fates in plants: (i) they can remain in the plastid and be involved in the synthesis of glycolipids especially galactolipids or (ii) they can be released to the cytosol and transfered to the endoplasmic reticulum to be used in extraplastidial lipid synthesis [116]. In microalgae, similar fatty acid trafficking could exist as studied in the diatom *Chaetoceros muelleri* with ^13^C-labelling [124].

After being synthesized in the plastid, saturated fatty acids are transferred to the cytosol to be elongated and/or desaturated. The elucidation of fatty acid synthesis pathways in microalgae is still ongoing. Even if a synthesis pathway of some species such as the diatoms are more extensively studied [107,125,126,127], for other species the synthetic routes remain to be identified. The following paragraph describes the polyunsaturated fatty acid synthesis pathway as it has been assumed to occur in plants and microalgae [116]. The conventional elongation and desaturation pathway present in numerous eukaryotes names, hereafter n-3 and n-6 pathways and the polyketide synthase pathway (PKS pathway), also exist to produce polyunsaturated fatty acids.

**Figure 2:**
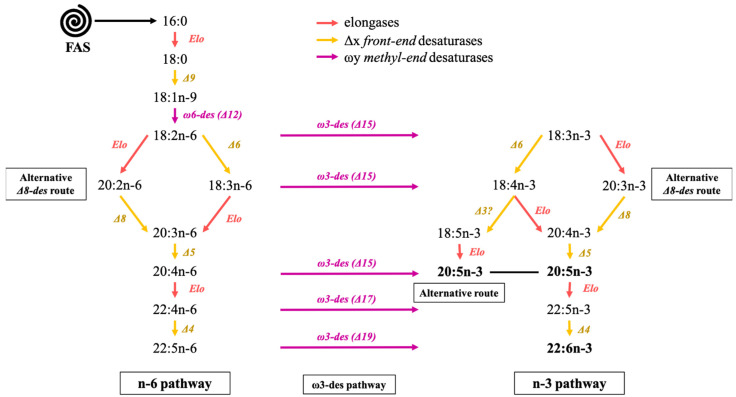
Fatty acid synthase pathway (FAS pathway) assumed for microalgae. Enzymes involved in desaturation and elongation processes from the conventional pathway are colored: yellow for front-end desaturases, magenta for methyl-end desaturases and red for elongases. Enzyme names are written next to or above the corresponding arrow.

Both n-3 and n-6 pathways are reported to performed in the mitochondria and the endoplasmic reticulum (ER) and present a similar synthesis sequence as those of palmitate, and involve specific enzymes acyl-CoA derivatives (Figure 2). In the conventional oxygen-dependent pathway, 18:1n-9 is desaturated by Δ12 desaturase and further by a Δ15 desaturase to form linoleic acid and α-linolenic acid, respectively, the precursors of the n-6 and n-3 fatty acid families [128]. Exclusively in diatoms, 16:1n-7 can be transformed by Δ6 and Δ15 desaturations into more complex C16 PUFAs (16:2n-7, 16:2n-4, 16:3n-4 or 16:4n-1) [127]. It is called the “C16 PUFAs pathway”. The different reactions producing more complex fatty acids from 18:2n-6 and 18:3n-3 are available in Figure 2. In nature, several desaturases complexes have been identified. Front-end desaturases (noted Δx) add the double bond at position x from the carboxyl-end of the carbon chain, while methyl-end desaturase (noted ωy) introduces the double bond at position y from the methyl-end [121,129]. Metazoan cells, for example, contain front-end desaturases such as Δ5 and Δ6 desaturases but do not possess methyl-end desaturases like ω6 (Δ12) or ω3 (Δ15). Mammals can elongate and desaturase dietary 18:2n-6 and 18:3n-3 into C20-C22 PUFAs (20:4n-6, 20:5n-3, 22:6n-3) using their Δ4, Δ5 and Δ6 desaturase. Nevertheless, as previously stated, the conversion rate of 18:2n-6 and 18:3n-3 into essential C20-C22 PUFAs is generally insufficient and thus has to be supplied by the diet. On the contrary, microalgae have both methyl-end and front-end desaturases and thus play an important role in providing higher trophic levels with these essential compounds.

### 5.4. Polyketide Synthase Pathway (PKS)

Another oxygen-independent pathway exists to synthesize unsaturated fatty acids and is called the polyketide synthase pathway, or PKS pathway [128]. In contrast to n-3 and n-6, which are more widely represented among microalgae taxa, the PKS pathway is limited to only some families, namely bacteria but also Thraustochytrids or Dinophytes [130,131]. The PKS pathway and FAS pathway share functioning similarities and rely on the same four basic reactions, namely condensation, reduction, dehydration and reduction by the four same enzymes: β-ketoacyl-ACP synthase (KS), β-ketoacyl-ACP reductase (KR), β-hydroxyacyl-ACP dehydrase (DH), and β-enoyl-ACP reductase (βER). While FAS synthesis and elongation/desaturation pathways, involving numerous enzymatic activities and reactions are very energy-consuming [132], the PKS pathway, in constrat, has fewer reduction and dehydration steps and can produce PUFAs more efficiently [133]. Indeed, the metabolites used to synthesize the fatty acid remain unsaturated as it gets lengthened. The PKS pathway, even if fundamentally anaerobic, can take place in aerobic conditions [134]. The PKS pathway was first identified in the deep-sea bacteria, *Shewanella* [133]. Later on, the discovery of PKS encoding genes as well as the identification of PKS routes in Thraustochytrids [135,136], Dinophytes [137,138,139], Haptophytes [140,141], and Chlorophytes [140] supported the broader existence of this pathway among protists. 

The polyketide synthases are subdivided into three types. Type I PKS are large multifunctional enzymes with several catalytic domains located on the same polypeptide. Each module is responsible for a set of distinct, non-iteratively acting activities responsible for the catalysis of one cycle of polyketide chain elongation [142]. Type II PKS are complexes composed of monofunctional enzymes, where each enzyme carries one catalytic domain and a single set of iteratively acting activities [142]. Finally, Type III PKS are homodimeric enzymes essentially involved in condensation reactions [142]. Type I and Type II need an acyl carrier protein (ACP) to activate the substrate responsible for the channeling of the growing polyketide intermediates. In contrast, Type III acts directly on the acetyl-CoA independently of ACP [142]. Type I PKS is mainstream of protist and has been identified in Haptophytes [141,143], Chlorophytes [140], Dinophytes [137,144], and Thraustochytrids [145]. In Dinophytes, the multifunctional activities characteristic of Type I PKS is not always verified because some species like *Karenia brevis*, *Alexandrium ostenfeldii,* and *Heterocapsa triquetra* possess an atypical architecture for Type I PKS with only one catalytic module [144,146]. Type II PKS is found in Haptophyte such as in the coccolithophorid *Emiliania huxleyi*, in Cryptophyte, and in Dinophyte [145]. Type III PKS is usually restricted to higher plants, fungi and bacteria [147,148].

**Figure 3:**
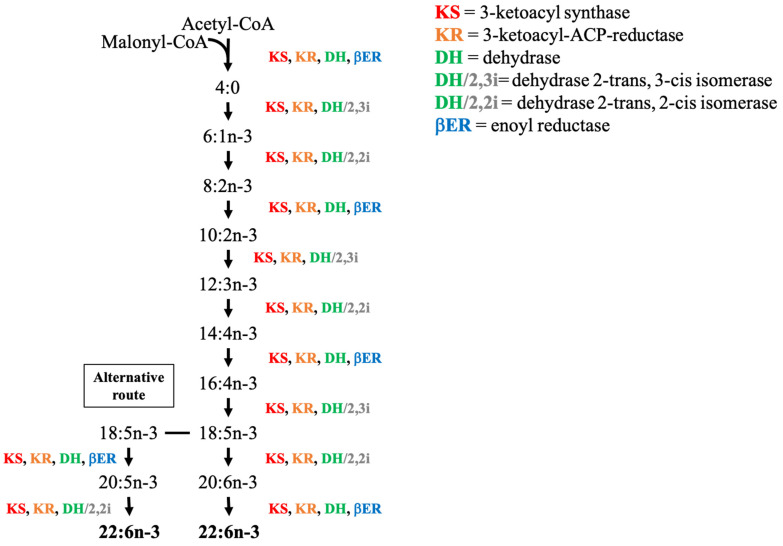
Polyketide synthase (PKS) pathway assumed for microalgae. Enzymes involved in the PKS pathway are the 3-ketoacyl synthase (KS—red), 3-ketoacyl-reductase (KR—orange), dehydrase with associated isomerases (DH—green, i—grey) and enoyl-reductase (βER—blue).

As mentioned earlier, the PKS pathway relies on the same four enzymes as the FAS pathway and can be completed with two isomerases (2.3 I or 2.2 I) (Figure 3). The precursors of the PKS pathway are identical to those of the FAS pathway i.e., acetyl-CoA and malonyl-CoA. The ketoacyl-synthase (KS) and ketoacyl-reductase (KR) are in charge of the addition of two carbon units while the dehydrogenase (DH) (or dehydrogenase associated with the isomerases) and enoyl-reductase (βER) are inserting the double bond. The difference between the functioning of PKS and elongation/desaturation pathways resides in this double bond insertion: the PKS pathway creates fatty acid by adding carbons and directly placing the unsaturation on a nascent acyl chain, while in n-3 and n-6 pathways, the double bond is inserted into an intact acyl chain [118]. Consequently, the PKS pathway requires less energy, as the ATP needed during the desaturation steps of the conventional pathway is not needed [118]. The different precursors and fatty acids assumed to be involved in the PKS pathway and proposed by Bell and Tocher (2009) [118] are presented in Figure 3. The large majority of the studies working on PKS have given a lot of attention to production of n-3 PUFAs, especially 22:6n-3 (DHA) and 20:5n-3 (EPA) [118,149]. However, more recent works show that PKS might not only be limited to n-3 PUFAs. Indeed, Zhang et al. (2017) [150] suggested the existence of a PKS-like pathway synthesizing the n-6 PUFA DPA-6, and Remize et al. (2020) [139] studied the fatty acid synthesis pathways of *Alexandrium minutum* and also proposed a n-6 PKS pathway to produce 22:5n-6. However, the difficulty in studying these pathways results from the fact that precursors used to form longer n-3 and n-6 PUFAs are generally absent, leading to further challenges in elucidating their synthesis routes [151].

### 5.5. Elucidation of Microalgae Synthesis Pathways

To better understand the pathways used by microalgae to synthesize fatty acids, several studies have focused on the elucidation of synthesis pathways using different techniques. While certain taxa, such as diatoms like *Phaeodactylum tricornutum*, are studied more extensively, other groups remain to be understood. Preliminary work used radioactive label ^14^C-acetate to study *P. tricornutum* synthesis pathway [107,125]. Using ^14^C-labeled substrate they showed that 20:5n-3 in diatoms might use a combination of both n-3 and n-6 pathways and that C_18_ PUFAs of the n-6 family (18:2n-6 and 18:3n-6) can be used to form corresponding C_18_ PUFAs of the n-3 family 18:3n-3 and 18:4n-3, respectively, by a ω3-desaturase. More recent works have focused on understanding gene expression. Indeed, genes are interesting targets for enhancing fatty acid metabolism and improving the production of DHA and EPA [152,153]. The discovery of new genes or new enzymes involved in these pathways could also guide research and help find new objectives. Consequently, new genes involved in the n-3 and n-6 conventional pathways or PKS pathway have been discovered in *P. tricornutum* and *Karenia mikimotoi* (Dinophyte), respectively, and could be used in understanding the selected routes for EPA and DHA synthesis [138,154].

Comprehension of these pathways, however, remains incomplete. More recent studies have thus focused on characterizing the metabolic pathways quantitatively. To do so, ^13^C-labelling flux analyses appear to be a promising approach and have already been used on microalgae [124,139,155]. While relying on an isotopically labeled substrate, this technique aims to monitor the label’s progressive incorporation into organic molecules of interest. This will identify the metabolic intermediates and end products, giving insights into biosynthesis pathways. Thanks to the development of a new generation instruments such as gas chromatography with mass spectrometry (GC-MS) or gas chromatography coupled to isotopic ratio mass spectrometry (GC-c-IRMS), considerable improvements have been made, and it is now possible to resolve the isotopic composition of organic macromolecules including fatty acids [156,157].

In the future, further understanding of microalgae polyunsaturated fatty acid synthesis pathways is necessary. The genes and enzymes involved and the identification of metabolic intermediates will be of central interest to increase polyunsaturated fatty acid production and to ease the development of valuable ingredients for food and feed industries.

## 6. Use of n-3 Polyunsaturated Fatty Acids in Food and Feed Industries 

### 6.1. Use and Proprieties of n-3 Polyunsaturated Fatty Acids in the Food Industry

Microalgae have been used in the human diet for centuries, especially in Asia and in Africa for species such as *Spirulina* sp. [4]. Thanks to their healthy and bioactive compounds such as carotenoids, phycobilins, fatty acids, polysaccharides, vitamins, and sterols, and also to their capability for production, they have been widely used in the health food market [4]. They have a range of relevant properties that can meet the increasing demand for sophisticated products [158]. The most popular way to consume microalgae is as a dietary supplement in tablet, capsule or powder form [159]. Indeed, the addition of the entire microalgal biomass is not always easy because of its color, fishy taste, smell and consistency [4]. An interesting approach in using microalgae as food is to employ the biomass as a source of specific biomolecules as it has already been tested in food and nutraceutical formulations [159]. Numerous studies have focused on extracting bioactive molecules and compounds such as fatty acids, proteins, pigments or antioxidants as additives for the food industry and as alternatives to the usual molecules [160,161,162,163].

The microalgal lipids can be divided into two major groups: (i) the neutral lipids (non-polar lipids) such as sterol, triacylglycerol (TAG), and free fatty acids are storage lipids and used as a stock of energy, and (ii) the polar lipids are structural lipid usually found in cell membranes. Short-chain saturated fatty acids (mainly neutral lipids) are valorized in the biofuel industry while, thanks to their health benefits, long-chain polyunsaturated fatty acids (esterified with glycerol in microalgae and generating TAG or polar lipids) are used in the nutraceutical industry or in active drug processing [164,165].

Microalgae biomass is predominantly utilized in the health food market and represents 75% of the annual microalgal biomass production [166]. Powders rich in n-3 PUFAs are manufactured into capsules or tablets and are expected to ensure a stable industry in the future [158]. This is the case for PureOne^TM^, a supplement capsule composed of EPA and DHA [165]. Allmicroalgae Natural Products (Portugal) developed health-benefits capsules and tablets with *Chlorella vulgaris* that are rich in omega-3, proteins, fiber, vitamins and iron under the brand Allma (known as Easy Capsules and Easy Tablets) [167]. Microalgae n-3 PUFAs are also formulated as oils with unique characteristics and different from plant or animal oils where oil is usually composed of a range of saturated and unsaturated fatty acids [130]. For example, Algae Omega 3 is formed with *Schizochytrium* sp. fatty acids and has been approved for human consumption [165]. OmegaTech (USA) exploits *Schizochytrium* to produce a low-cost oil known as DHA Gold [168,169]. This oil is currently supplemented as an adult dietary supplement in food (like cheese, yogurts, spreads and dressings) and beverages [168]. Nutrinova (Germany), and Martek (USA), respectively, cultivate the microalgae *Ulkenia* sp. (Thraustochytrid) and *Crypthecodinium cohnii* (Dinophyte) to produce similar oils rich in DHA [130].

*Chlorella* has been cultured in large-scale structures for the production and commercialization of fatty acids (including omega 3 and omega 6) because it has been recognized for its stimulation properties for the human immune system [167,170]. The green edible microalga, *Dunaliella salina*, a by-product of large-scale salt producing evaporative ponds, is also valorized as health food [171,172]. Babuskin et al. (2014) [173] used *Nannochloropsis oculata* for its richness in omega-3, EPA and DHA, to create functional cookies and pasta with health benefits. *Pavlova lutheri* was supplemented in the yogurt formula for its n-3 PUFA content having anti-inflammatory proprieties in vitro which can also be interesting for consumer health [174]. However, the algae-based yogurt was not pleasant enough for consumers and needed improvement [174]. Incorporating enriched n-3 PUFAs algae oils into yogurt seems like an interesting idea to consider for new food development, especially for vegetarians [175]. Ingestion of this enhanced-yogurt significantly increases the level of DHA in the plasma and blood erythrocytes [175]. Consequently, another advantage of using algae to synthesize these beneficial compounds for humans also resides in the fact that they seem an appropriate ingredient for vegetarians [175]. Species like *Schizochytrium* sp. presented before and recognized for their high DHA levels are more and more consumed for their DHA-rich oil and are also suitable for a vegetarian diet [169]. They are also particularly interesting as they are known to be safe, without detectable environmental pollutants or heavy metals [169].

Vegetarians who supplemented with DHA from *Ulkenia* sp. showed positive results on the accumulation of n-3 PUFAs of interest in red blood cells. This tested microalga oil also seemed to impact their appetite and regulation of food intake [176]. N-3 PUFAs (EPA, DHA, ALA) can be supplemented in health drinks, such as infant grade milk or in *Chlorella*-enriched beverages [158].

Microalgae lipids can be used for their antioxidant, anti-cancer and anti-inflammatory properties. For example, furan fatty acids are free radical scavengers and present an electron-rich furan ring within the carbon backbone [177]. They have been identified as powerful anti-inflammatory agents and are known so far in *Isochrysis* sp. and *Phaeodactylum tricornutum* [177,178,179]. Their development and use in the food industry are still in their infancy, as no clinical trials have been performed to evaluate their effect on cardiometabolic health because they are difficult to extract and purify [177]. The key link between n-3 PUFA and inflammation is eicosanoid metabolism [30]. Indeed, eicosanoids are mediators and regulators of inflammation and are generated from C_20_ PUFAs [30]. Nitrogen-containing compounds (lipopeptides) produced by cyanobacteria aim for tubulin or actin filaments in eukaryotic cells allowing their detection by anticancer agents [180]. Phytosterols containing food can also be interesting in reducing the concentration of blood cholesterol and preventing the onset of cardiovascular diseases, diabetes and hypertension [166].

However, microalgae PUFAs’ functionality in food products is not limited to their health aspects, as they can also have bioactive properties that can be used in food formulation, for example, in structuring processes [12]. In the following paragraph describes examples of the application of microalgae lipid fractions in the food industry.

Species rich in proteins such as *Chlorella* sp., *Haematococcus pluvialis* or *Tetraselmis* sp. were studied as promising microalgae for the production of surface-active ingredients [181,182]. Indeed, the complex structure of proteins makes them particularly suitable for production of stable viscoelastic film valuable in emulsion [183,184]. However, Law et al. [184] stated that proteins might not always be as good of a surfactant, i.e., reducing interfacial tension between oil and water phases despite being an excellent emulsifier as smaller molecular weight molecules especially in food processing. Indeed, small molecular-weight surfactants have a higher diffusivity and are more easily available to absorb and lower interfacial tension [183]. Consequently, small molecular-weight compounds produced by microalgae such as lipids, have been studied for their surface-active proprieties. This is the case of glycolipids (monoacylglycerol—MAG or diacylglycerol—DAG), phospholipids (phosphatidylcholine—PC also called lecithin) or fatty acids [185,186,187]. Marine phospholipids contain high levels of lecithin (phosphatidylcholine—PC) which have amphiphilic properties, and thus are particularly suitable for emulsion preparation [188]. Most of the current literature on the subject discusses the use of n-3 triacylglycerol fortified functional foods, whereas food fortification with phospholipids is less studied [188]. For example, n-3 PUFA phospholipid rich oils have been used to fortify surimi seafoods and confer them a better resistance towards lipid oxidation due to the presence of EPA and DHA [189]. Similar types of phospholipids have been used to create nutraceutically fortified egg products [190,191].

Microalgae biomass and lipids fraction are also used as food quality enhancers and preservatives. The development of algae-based lipid powders and flours is a trending topic and is more and more included in novel cuisine. The addition of the edible *Dunaliella salina* to pasta recipes increases its sensory characteristics especially due to the higher presence of polyunsaturated fatty acids, phytochemicals, and minerals [192]. They are being used as egg substitutes with really promising results, especially for vegetarians [193]. Lipid powders, especially those rich in 14:0, 16:0, 18:1n-9 and EPA from microalgae, are also interesting for their antimicrobial properties, which can play a significant role in product preservation [193]. Microalgal biomass is used to increase the nutritional properties of dairy food products such as cheese. *Chlorella* and *Arthrospira* have been used in cheese and reported to positively impact lactic acid bacteria viability and enhance quality and sensory properties [194]. *Nannochloropsis oculata* and *Isochrysis galbana* were used in novel cookies, pasta and biscuit formulas and significantly increased the concentration of n-3 PUFAs inducing a positive sensory evaluation by consumers [173,195]. *Auxenochlorella protothecoides* were used to create an algal flour (AlgaVia^®^ Lipid-Rich Whole Algae by Corbion) rich in polyunsaturated fats be used in various types of foods [194]. Another important advantage in adding microalgae biomass rich in lipids, especially n-3 PUFAs, could be their higher resistance to thermal treatment [195,196]. This preserves the greater nutritional quality of the microalgae-enriched-products in comparison with conventional products.

### 6.2. Example of Microalgae Valuable in Food Industries

As previously mentioned, only a few microalgae species are intensively used for human consumption, among them *Chlorella vulgaris*, *Spirulina* (*Arthrospira*), and *Tetraselmis chuii*; nevertheless, *Dunaliella*, *Haematococcus*, *Schizochytrium*, *Scenesdesmus*, *Aphanizomenon*, *Odontella,* and *Porphyridium* are gaining acceptance in the food and health food market [197]. Currently, *Chlorella* and *Arthrospira* largely dominate the market [158]. Allmicroalgae, based in Portugal, is commercially supplying the species approved for human consumption in powder and frozen paste format to be added to soups, millets, juices, crackers, cookies, ice creams, smoothies or dietary supplements [198]. Indeed, *Chlorella vulgaris* and *Arthrospira* are relatively easy to grow. *Arthrospira* grows in an alkaline environment and under saline conditions that prevent the growth of competitive organisms or contaminants [199]. Cyanobacteria are mainly sold in Europe, North America, and Asia [168]. *Spirulina* (*Arthrospira*) has been used for years, especially by African and Mexican populations living around alkaline lakes where it develops naturally [200]. Although mainly used for its richness in polysaccharides and phycobiliproteins, *Arthrospira* is also known for its important production (3–35%) of the essential γ-linolenic acid (18:3n-6—GLA), which is pharmacologically significant as it is effective in lowering plasma cholesterol and in stimulating prostaglandins [198,200]. However, it can also synthesize DHA in lower proportions (9% of total fatty acids content) that is involved in regulation of inflammatory, immunological, and cardiovascular disorders [198,201]. *Chlorella* has been widely cultivated and commercialized in the health food market [199]. *Chlorella* is a spherical unicellular green alga that reproduces asexually [202]. One advantage of this species is its capacity to be cultivated in heterotrophic, autotrophic, and even mixotrophic conditions [199]. Its composition in polyunsaturated fatty acids (especially 18:2n-6 and 18:3n-6) has been shown to confer antioxidant, antitumor and anti-inflammatory proprieties valued in the food industry [201,203]. Chlorophytes generally contain low levels of EPA, with an exception made for the species *Chlorella minutissima,* that has been proven to produce a high content of this LC-PUFA in photoautotrophic conditions (31% of total fatty acids) or when incubated at low temperatures (45% *w*/*w*) [204,205]. *Chlorella* has been used for health food production in Germany, China, Japan, and other Asian countries and valorized as food supplements for humans [202]. *Tetraselmis chuii* (Chlorophyte) is another species valorized in food and health products. In Europe, *T. chuii* has been approved as a novel food to be used as a sauce, special salt, and condiment since 2004 [194], while the Thraustochytrid *Ulkenia*, rich in DHA, was approved in 2009 as a novel food ingredient in bakery products, cereal bars, and nonalcoholic beverages [206]. Other species such as *Crypthecodinium*, *Nannochloropsis*, *Phaeodactylum*, *Monodus*, *Nitzschia* or *Isochrysis,* are currently under investigation for their health supplement, especially their PUFA production [207]. In the United States, the Food and Drug Administration (FDA) ranked microalgae as generally recognized as safe (GRAS). This is the case for species such as *Euglena gracilis* for conventional food and beverage products such as baked goods, cereals, milk and dairy products, processed fruits, fruit juices, soft candy, and soups [194]. Other GRAS species are *Arthrospira platensis*, *Auxenochlorella protothecoides*, *Dunaliella bardawil*, *Dunaliella salina*, *Schizochytrium* sp., and *Ulkenia* sp. [194,208]. Microalgae species are authorized in Europe, France and the USA, and examples of applications are available in Table 1.

### 6.3. Use of n-3 Polyunsaturated Fatty Acids in the Feed Industry and Aquaculture

Microalgae are also used for feed, especially in aquaculture, since they represent the conventional diet of marine and freshwater consumers in natural ecosystems [179,198]. Species like *Dunaliella*, *Chlorella*, *Arthrospira*, *Nannochloropsis* or *Tetraselmis* proved to have good application in aquaculture [1]. Indeed, species like *Nannochloropsis* sp. are characterized by high levels of PUFAs (37%) and are rich in EPA and DHA [217]. Chlorophytes such as *Chlorella*, generally have low nutritional values due to their low levels in essential long-chain polyunsaturated fatty acids, and are then often combined with other species when used as feed [218]. The Baccilariophyceae, including diatoms (such as *Chaetoceros* sp. or *Phaeodactylum* sp.), are more interesting as they produce important levels of EPA [111].

Microalgae are cultivated and concentrated in the form of dispersible pastes (which can be added directly in the water tank and dissolved in the liquid media) or as freeze-dried cubes [198]. When used alive, microalgae are a source of essential fatty acids [198]. To form the microalgae pastes, diatoms such as *Chaetoceros* sp. and *Phaeodactylum* sp. or the Eustigmatophyte, *Nannochloropsis oculata* are generally more used [198]. As for food industries, microalgae rich in PUFA can be used as functional feed. Microalgae biomass can enhance animal physiology by improving their immune response, disease resistance, antiviral and antibacterial protections, and better reproductive performance, feed conversion, and weight gain [219]. In the domain of aquaculture feeding, microalgae biomass appears really promising for a more sustainable animal production industry as they are more environment-friendly than classical land agriculture [219].

Mollusk species are the largest aquaculture group of animals that rely on microalgae as feed [179]. As an example, in 1999, 62% of the microalgae produced for aquaculture were used for mollusks, while 21% were used for crustaceans (mainly shrimps) and only 16% for fish [168]. Microalgae species such as *Isochrysis* affinis *lutea* (T-iso), *Pavlova lutheri,* and *Chaetoceros* sp. are commonly used in shellfish hatcheries for their richness in nutrients, especially n-3 PUFAs [199]. They are easily and efficiently filtered from the water where the shells grow [179]. Crustaceans also feed directly on microalgae, and their PUFAs are essential during the larval stage [168,220].

Microalgae can be used for their suspected role as probiotics. This remains to be proven, however, and concrete evidence of the action of living microalgae in aquaculture farms is still scarce [219]. For example, *Chaetoceros* sp., *Pavlova* sp., and/or *Isochrysis* sp. have been added to pearl oyster diets and have been shown to improve their resistance to bacterial pathogens [221].

Microalgae can also be utilized indirectly in aquaculture feed. Indeed, heterotrophic protists and small zooplankton (such as rotifers of genus *Brachionus* or the brine shrimp *Artemia salina*) play a major role in supplying and channeling energy and essential compounds from microalgae to higher consumers [222,223]. By feeding on microalgae, these organisms give access to nutrients, polyunsaturated fatty acids, amino-acids, and sterols to higher trophic levels that are originally unable to use them due to their small sizes [223,224]. For example, the heterotrophic dinoflagellate *Crypthecodinium cohnii* has been marketed for its DHA production and used as a substitute for fisheries-derived oils for seabream (*Sparus aurata*) microdiets [225]. The elevated DHA production of *Crypthecodinium* was particularly suitable for the high requirement for this essential PUFA of seabream larvae and resulted in similar performances as with classical fisheries-related diets [225]. *Nannochloropsis* sp. and *Isochrysis galbana* are also widely used for rotifer production and to enhance their richness in EPA and DHA [226,227,228]. Indeed, they increase their survival, productivity, efficiency of feed assimilation, and biochemical composition [227,228]. *Schizochytrium* can also be supplemented to zooplankton rotifers to increase their levels of DHA [229]. Special care has to be given to the microalgae’s culture condition because they can have a significant effect on their nutritional quality for predators, especially since rotifer fatty acid composition is closely related to that of their diet [228,229]. Rotifers enriched with high lipids levels can be used as the first food organisms in culture of fish and shrimp larvae [226,229]. They are in charge of supplying consumers with macro- and micronutrients, vitamins and even antibiotics [226]. An advantage of using lipids from microalgae for aquaculture is the possibility of adjusting and enhancing the nutritional composition of microalgae by monitoring environmental parameters [73].

Microalgae can also be incorporated into the feed of terrestrial animals and positively impact their survival, development, growth, and fertility [199]. Species like *Spirulina*, *Chlorella*, *Tetraselmis*, *Nannochloropsis*, *Nitzschia*, *Navicula*, *Scenesdesmus*, *Crypthecodinium,* and *Chaetoceros* are valorized and commercialized for pets, ruminants, pigs, poultry and other animals [230]. *Arthrospira* is also highly used in this domain and touches all types of animals, such as cats, dogs, aquarium fish, ornamental birds, horses, cows, and breeding bulls, because it affects their physiology to essential fatty acids among other [168]. *Crypthecodinium cohnii* and *Schizochytrium* sp. are used as poultry feed, especially for chickens to produce eggs rich in omega-3 [165]. As for humans, EPA and DHA have been credited with health benefits even when supplemented in small amounts, and can have positive effects on the improvement of the immune system, lipid metabolism, stress resistance, increase in appetite, enhancing reproductive performance, or even the reduction of cholesterol levels [231]. *Nannochloropsis oceanica* has been tested as a natural source of EPA for ruminants [232]. The algae’s thick cell walls protect the healthy compound from metabolization by rumen microbiota, inducing a full enrichment in the EPA for better ruminant development and health without inhibiting the growth and activity of rumen bacteria [232]. Providing n-3 PUFAs has a positively impact on atopic dermatitis, osteoarthritis, and the modulation of immunity indicators in dogs [233]. DHA in dog diets can improve their immune system, enhance the palatability of their food, increase digestibility, and enhance their cognitive responses [234,235]. The use of Algal Oil rich in EPA and DHA has been proven efficient in Beagle dogs during their reproduction, development, growth, and during the early life stages of their offspring [236]. In shrimp hatcheries, microalgae are necessary for their PUFA production that participates in the second stage of larval development (zoea) [218].

Consequently, industrial companies are progressively developing feed for pets, poultry and cattle based on microalgae biomass or extracts to improve their health or a better nutritional quality. This is the case, for example, with Allvitae by Allmicroalgae Natural Products, which propose various ranges of dog, cat, swine, bird, and poultry feeds rich in omega-3, especially DHA from species such as *Chlorella vulgaris*, *Tetraselmis chui*, *Nannochloropsis oceanica*, *Scenesdesmus obliquus* [237]. These feeds enhance their vital functions, heart conditions, cognitive development, or stimulate their intestinal immunity [237]. Veramaris proposes a feed (Veramaris^®^ *Pets*) rich in EPA and DHA via the use of algal oil and used for aquafeeds. *Schizochytrium limacinum* is used to made All-G-Rich^TM^ (Alltech), a dehydrated whole-cell meal for chicken composed of 16% of DHA [238]. Hens supplemented with All-G-Rich can produce eggs richer in DHA without modifying their quality [238]. AlgaPrime^TM^ from Corbion is also marketed for animal feed and consists of a DHA source from *Schizochytrium* sp. [239].

## 7. Conclusions

EPA and DHA are receiving increasing interest for their positive impact on human health thanks to their antioxidant, anti-inflammatory and anti-cancer properties. Originating mainly from marine sources such as fish, these two compounds face supply challenges due to current stock reduction and overfishing. Because of environmental stress and increased pressure on the natural stock, aquaculture is not sufficient for filling the gap between supply and demand. New techniques, such as genetic engineering to increase the production of these molecules for humans, are in development.

As primary producers of EPA and DHA, microalgae have been used in recent years to develop the future ingredients and foods. Currently, only a small proportion of known microalgae species are used in the food and feed industry, as authorization proving they are safe for consumption is difficult to obtain. A rising number of companies are using n-3 PUFA as a functional ingredient for health food, food, supplementary food, or even for their physicochemical properties. The nutritional quality can enhance the final product for humans and both marine and terrestrial animals.

In the future, increasing the application of these healthy compounds is expected to and must be associated with increased knowledge of microalgae synthesis pathways to improve their production sustainably. Authorities should also allow more microalgae to be used in this sector to expand the range of possibilities.

## Figures and Tables

**Table 1:** Microalgae used in the food industry and authorized in France, Europe, and the USA (GRAS: generally recognized as safe), and examples of application. DS: dietary supplement, E: extract, EP: Europe, F: Food, FR: France, USA: United States of America.

Scientific Name	FR [209]	EP [209]	USA [194,208]	Example of Application
*Aphanizomenon flos aquae*	DS	F		Functional ingredient with antioxidant properties for cookies [210]
*Arthrospira* sp.	F	F	DS, F	Fermenting agent for lactose-free beverages [211]
*A. major*	DS			
*A. maxima*	DS			
*A. platensis*	DS	F	DS, F	
*Auxenochlorella protothecoides*			DS, F	Algal flour for baked goods [194]
*Chlamydomonas reinhardtii*			DS, F	Omega-3 fatty acids valuable in healthy food [212]
*Chlorella* sp.	F			Tablets and powder made with whole biomass for human food [167]
*C. luteoviridis*		F		
*C. pyrenoidosa*		F		
*C. vulgaris*	DS	F	DS, F	
*Dunaliella salina*	DS		DS, F	Biomass used in pasta [192]
*D. badarwil*			DS, F	
*Euglena gracilis*			DS, F	Produce paramylon acting against fatigue [213]
*Haematoccocus pluvialis*	DS	DS, E		Astaxanthin-rich oleoresin with antioxidant capacity for healthy food [214]
*Nannochloropsis oculata*	DS			Ingredient in functional cookies [173]
*Odontella aurita*	DS	DS		Produces fucoxanthin with antioxidant activities [215]
*Schizochytrium* sp.	DS	E	E	Used for DHA-rich oil [165]
*Tetraselmis chuii*		DS, E		Ingredient for broccoli soup [216]
*Ulkenia* sp.	DS	E	E	Used for DHA-rich oil [130]
